# One Possible Consequence of COVID-19 Vaccine: Inequitable Distribution

**DOI:** 10.1093/geroni/igab046.1227

**Published:** 2021-12-17

**Authors:** George Garrow

**Affiliations:** Primary Health Network, Sharon, Pennsylvania, United States

## Abstract

Primary Health Network (PHN) is the largest Federally Qualified Health Center (FQHC) in Pennsylvania expanding over 17 counties. Getting Pennsylvanians vaccinated is a critical step in reducing the spread and impact of COVID-19, although research suggests that the inequitable distribution of the COVID-19 vaccine may be a critical barrier. Although concerns regarding vaccine hesitancy are prevalent, experts also suggest that disparities in vaccination rates are in part due to the lack of accessible scheduling; adversely affecting underserved, such as rural communities, and minority populations. To address these obstacles, Primary Health Network is creating a COVID-19 Vaccination/Health Equity Team. Their objectives include: creating tools to provide comprehensive information on vaccine supply, identifying potential challenges and proactively planning for ways to mitigate likely disparities, identifying people who wish to be vaccinated but lack the means to do so, and connecting them in an equitable way, to vaccinations.

